# Eliminate all risks: A call to reexamine the link between canine scabies and rheumatic heart disease

**DOI:** 10.1371/journal.pntd.0012115

**Published:** 2024-05-02

**Authors:** Victoria J. Brookes, Caitlin E. Henning, Kate A. Worthing, Chris Degeling

**Affiliations:** 1 Sydney School of Veterinary Science, Faculty of Science, The University of Sydney, Camperdown, New South Wales, Australia; 2 Sydney Infectious Diseases Institute, Faculty of Medicine and Health, The University of Sydney, Westmead, New South Wales, Australia; 3 Australian Centre for Health Engagement, Evidence and Values, Faculty of the Arts, Social Sciences and the Humanities, University of Wollongong, Keiraville, New South Wales, Australia; TOBB Economics and Technology University Faculty of Medicine: TOBB Ekonomi ve Teknoloji Universitesi Tip Fakultesi, TURKEY

## Abstract

Rheumatic heart disease (RHD) and acute rheumatic fever (ARF) disproportionately affect individuals in low-resource settings. ARF is attributed to an immune response to Group A *Streptococcus* (GAS) following GAS pharyngitis and potentially GAS impetigo in which infection can be initiated by scabies infestation. The burden of ARF and RHD in Aboriginal and Torres Strait Islander people in Australia is among the highest globally. Following recent calls to include dog management programs in ARF and RHD prevention programs, we believe it is timely to assess the evidence for this, particularly since previous recommendations excluded resources to prevent zoonotic canine scabies. While phylogenetic analyses have suggested that the *Sarcoptes* mite is host specific, they have differed in interpretation of the strength of their findings regarding species cross-over and the need for canine scabies control to prevent human itch. Given that there is also indication from case reports that canine scabies leads to human itch, we propose that further investigation of the potential burden of zoonotic canine scabies and intervention trials of canine scabies prevention on the incidence of impetigo are warranted. Considering the devastating impacts of ARF and RHD, evidence is required to support policy to eliminate all risk factors.

Rheumatic heart disease (RHD) and its precursor, acute rheumatic fever (ARF), disproportionately affect people in low-resource settings [1]. ARF is attributed to an immune response to Group A *Streptococcus* (GAS) following GAS pharyngitis and potentially GAS impetigo in which infection can be initiated by scabies infestation [2].

In 1999, it was stated that control programs for human scabies did not require resources directed against zoonotic scabies from dogs [3]. Twenty years later, the 2020 Australian RHD Endgame Strategy called for dog management programs (Fig 1) as part of primordial prevention (reducing the development of risk factors for disease) of RHD and its precursor, ARF [4]. In this viewpoint, we examine the background and current evidence for this change in recommendation. To develop evidence-based policy in line with the recommendations in the RHD Endgame Strategy, we find that there is sufficient indication that canine scabies causes human itch to warrant further investigation, including the effect of canine ectoparasite control on the incidence of GAS skin infections in people.

**Fig 1:**
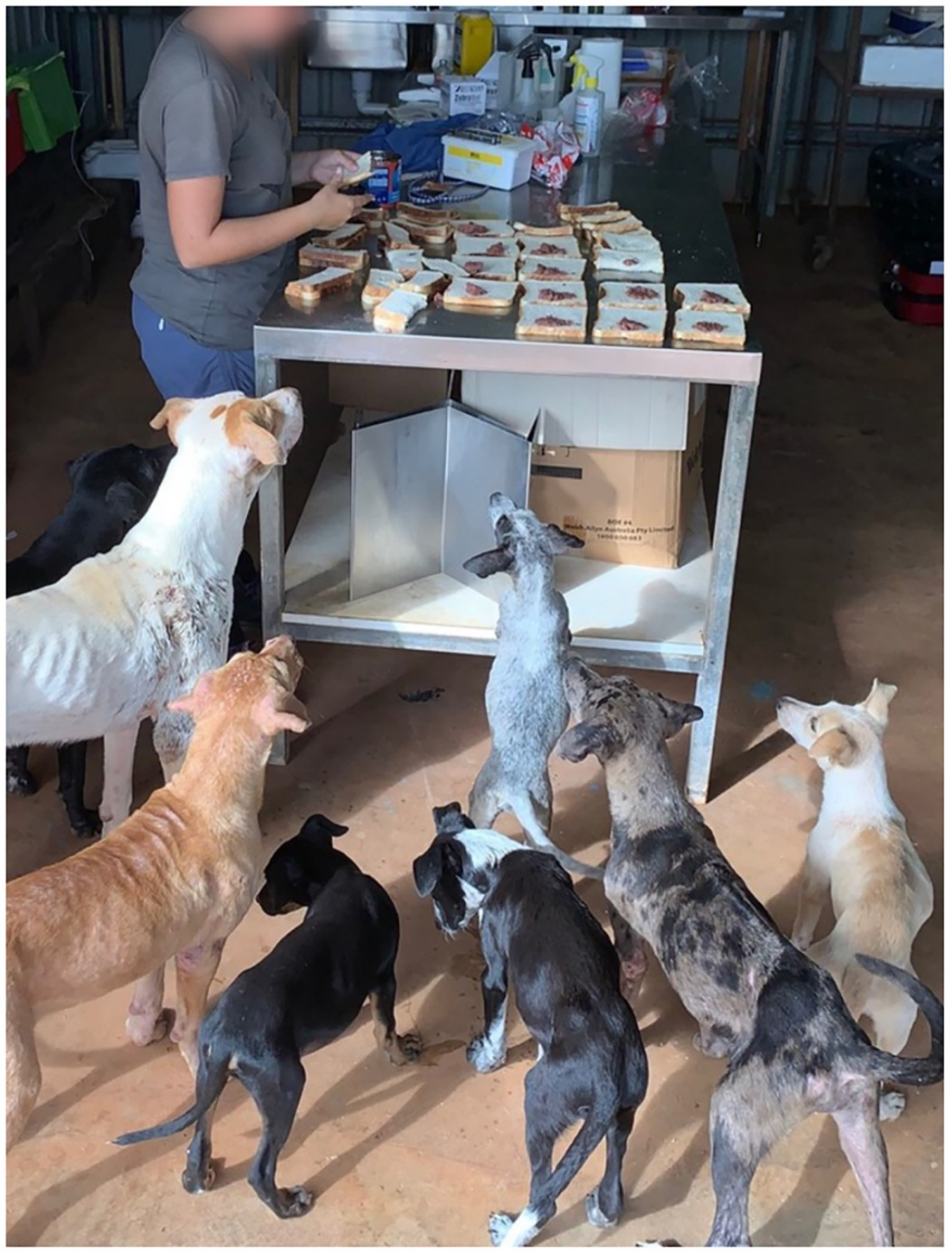
Dogs waiting for sandwiches containing parasiticide in a remote community in northern Queensland (photograph courtesy of *K*. *Smith and D*. *Smith*).

Because of the enduring impacts of colonisation, Aboriginal and Torres Strait Islander people in Australia face a significantly worse health burden than non-Indigenous Australians, with a lower life expectancy of approximately 8 years [5]. Among the most divisive diseases are RHD and ARF. In a national study from 2015 to 2017, age-standardised first-incident ARF rates were 124.1 times higher (95% confidence interval, 105.2–146.3) in Indigenous (71.9 cases/100,000 people) than non-Indigenous populations, and age-standardised RHD prevalence in Indigenous people was 61.4 times higher (95% CI, 59.3–63.5) than in non-Indigenous people [6]. Although ARF and RHD have historically been considered diseases of low- to middle-income countries, Aboriginal and Torres Strait Islander Australians—particularly children in remote communities—continue to face among the highest reported burden of ARF and RHD globally [7].

ARF is an autoimmune disease in response to infection with GAS, which typically manifests as arthritis, chorea, and carditis [2]. Progression to RHD follows cumulative damage to the heart valves with recurrent episodes of ARF, potentially leading to lifelong disability and premature death. A link between GAS pharyngitis and ARF is established, but throat carriage of GAS species is reportedly low in people in remote communities in Australia. In these settings, ARF is considered more likely to follow GAS impetigo although the pathogenesis is unclear [8].

In 2020, the RHD Endgame Strategy was introduced with the aim of eliminating RHD in Australia by 2031 [4]. RHD prevention and control following ARF conventionally includes long-term monthly injections of benzathine benzylpenicillin and cardiac ultrasonography and surgery, but the rigorous schedule of painful injections and barriers to healthcare access in remote communities frequently lead to poor outcomes. The new strategy focuses on primordial prevention by comprehensively addressing the social determinants of health to reduce risk factors associated with ARF and RHD [4]. Nine “Healthy Living Practices” (https://www.healthabitat.com/what-we-do/safety-and-the-9-healthy-living-practices/, accessed 2023 Jun 1) are included that promote healthy environments, such as climate and culturally appropriate housing that enables close living, with sufficient resources including health hardware (essential resources and infrastructure for healthy living, such as correctly installed electricity and plumbing in well-maintained kitchens, bathrooms, and laundries). Australian Aboriginal children have among the highest reported prevalence of impetigo globally [9] and the healthy living practices outlined in the strategy aim to reduce the risk of GAS transmission and infection.

Dog management programs are recommended as a component of “Healthy Living Practice 6,” which focuses on reducing the risk from animals, insects, and vermin [4]. However, in the RHD Endgame Strategy, it is stated that the benefits of dog management programs in reducing impetigo are anecdotal; there is no evidence that dogs are colonised by GAS, or that dogs transmit GAS to people [10,11]. Although dogs do not carry Group A *Streptococcus*, cross-species transmission of Group C *Streptococcus* between a child and a dog residing in the same household has been demonstrated [12]. The ability of other streptococci to infect both dogs and humans makes dogs a potential reservoir of virulence genes for GAS. Furthermore, dogs harbour skin parasites that can cause human itch, including fleas (usually the cat flea, *Ctenocephalides felis*) and the canine variant of the *Sarcoptes* mite [13,14].

Scabies, a highly pruritic skin condition caused in people by the mite *Sarcoptes scabiei* var. *hominis*, is a WHO-listed neglected tropical disease and endemic in many Australian remote communities, where it is considered an important risk factor for impetigo due to scratching and potential interference with skin immune defences [9,15–17]. The canine variant of the *Sarcoptes* mite, *Sarcoptes scabiei* var. *canis*, is also endemic to dog populations in many remote communities (Fig 2) [13]. There has been limited research into the zoonotic potential of the canine mite. This is perhaps due to 2 studies from 1999 and 2004 reporting genetic clustering of mites according to host species, concluding that prevention of zoonotic scabies from dogs was unnecessary [3,18]. However, 2 subsequent phylogenetic analyses have called into question whether *S*. *scabiei* mites originating from Australian people and dogs cluster into 2 distinct groups [19,20]. Firstly, Morrison (2005) used network phylogenetic analysis of the same markers examined previously and found that human and canine scabies mites were interspersed in the tree, suggesting that not only was there evidence for a transmission route between humans and dogs, but also the direction of transmission was from dogs to humans [19]. Morrison (2005) concluded that canine scabies should not be ignored in a human scabies control program. Secondly, Zhao and colleagues (2015) used the mtDNA *cox1* gene to construct a phylogenetic tree of human and animal isolates from several countries including China and Australia [20]. Crucially, while most human isolates from around the world formed clades that were distinct to country and species, some isolates from Australian humans clustered with the isolates from Australian dogs. The mtDNA *cox1* phylogenetic tree had bootstrap support of more than 97%, indicating that in 97% of repeat samplings, the tree branches would remain in the same location, indicative of a true phylogenetic clustering rather than one due to chance. In support of these findings, there are case reports of intensely pruritic infestations with the canine mite in people, albeit usually self-limiting, as well as zoonotic scabies from other companion animal species, livestock, and wildlife [21]. These reports and the findings of Morrison (2005) and Zhao and colleagues (2015) point to the need for further genomic studies, particularly focusing on the Australian population.

Dogs are an integral and important part of life for Aboriginal and Torres Strait Islander people in remote communities. Recent dog counts in northern Australian remote communities estimate 22 to 62 dogs/100 people, greater than the national average of 16 dogs/100 people, and most dogs are owned and free-roam [22–24]. Dogs have day-to-day roles as companions, protectors, and as hunters, and their cultural value is demonstrated in dreaming stories, totems, and—in some regions—inclusion in the kinship system [25–27]. Dog management programs, led by Environmental and Animal Health Workers (EHWs) are vital to maintain dog health and prevent overpopulation. The presence of dogs in Aboriginal and Torres Strait Islander communities have individual and community-level benefits, broadly enhancing human and dog wellbeing and intra-family connectivity [28].

**Fig 2:**
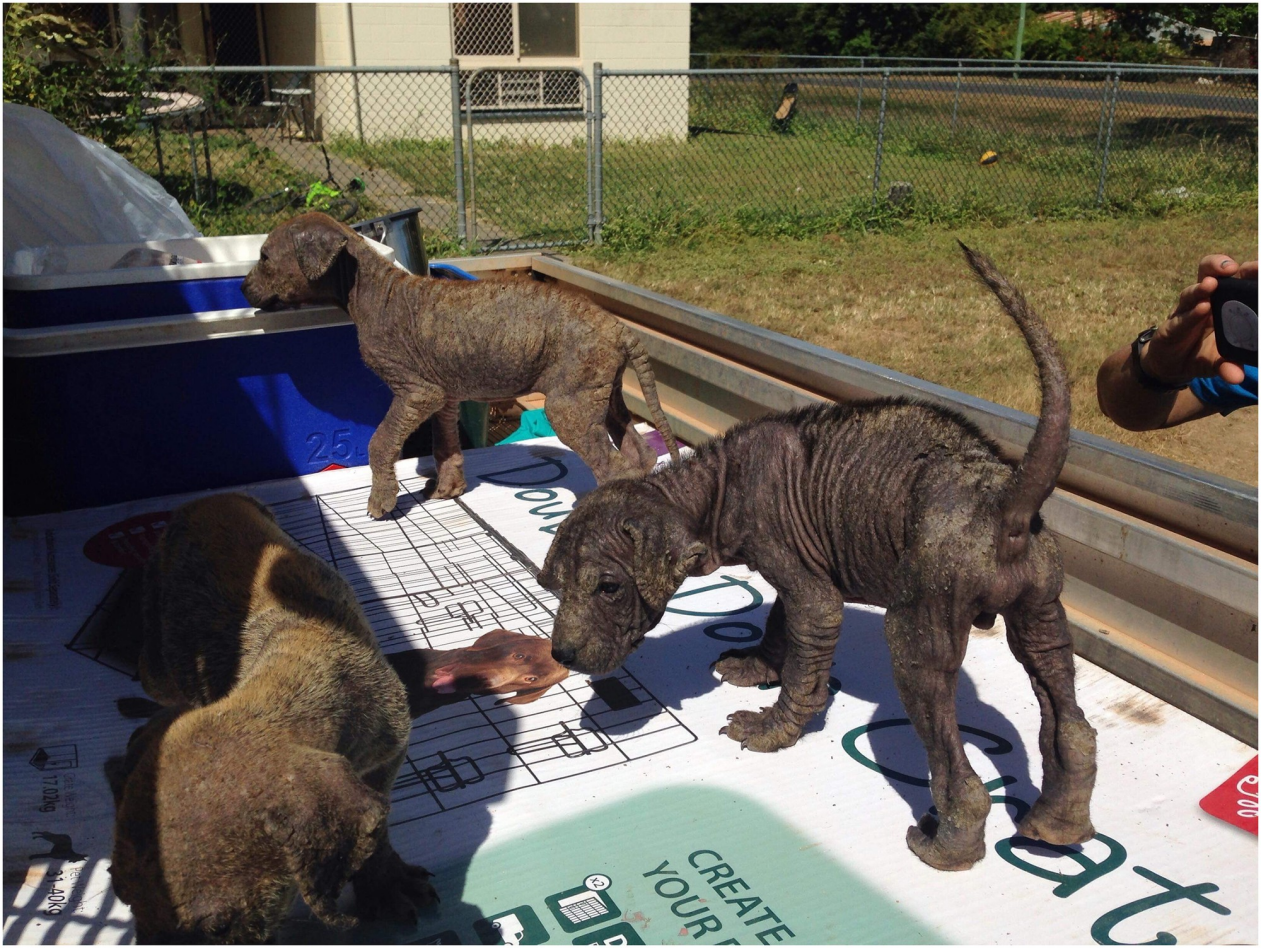
Owned puppies with skin disease due to scabies, in a remote community in northern Queensland (photograph courtesy of *K*. *Smith and D*. *Smith*).

Research has demonstrated that treating human scabies reduces impetigo incidence in remote northern Australian communities [8]. There have been no studies of the effect of canine ectoparasite control on impetigo occurrence in people. Canine parasite control is a routine part of dog management programs; however, program delivery can be sporadic due to insufficient funding [28]. Long-acting anti-parasiticides for dogs have recently been developed—including afoxolaner and fluralaner which have been shown to reduce mite burden in *S*. *scabiei* var. *canis* infestations in dogs [29,30]—which might offer options for consistent population-level control programs to eliminate canine scabies in dogs in remote communities.

A forecast released in 2018 predicted that if observed incidence rates of ARF and RHD remain stable, >5,000 Aboriginal and Torres Strait Islander people will be living with RHD and >650 will die by 2031, with associated medical costs >AUD300 million [31]. Given the current and expected burden of disease, an evidence-based approach should be taken to eliminate all risks. Case reports and phylogenetic analysis provide sufficient indication that canine scabies as a cause of human itch should be investigated further, and intervention trials to investigate the effect of canine ectoparasite control on the incidence of GAS impetigo in people are warranted. In addition, the pathogenesis from GAS impetigo to ARF still needs to be elucidated. If a pathway from impetigo to ARF is confirmed, and dog scabies is a source of itch leading to GAS impetigo in people, this would support evidence-based direction of resources towards dog management programs in remote communities in line with the RHD Endgame Strategy. Evidence is essential to enable comprehensive policy development towards preventing the devastating impacts of ARF and RHD.
